# Supplementary material for: Breast cancer cell-secreted miR-199b-5p hijacks neurometabolic coupling to promote brain metastasis
Source: Nat Commun. 2024 May 29;15:4549. doi: 10.1038/s41467-024-48740-0 (PMC11137082; doi:10.1038/s41467-024-48740-0)
Supplement: Supplementary file 1 — Supplementary Information [file 41467_2024_48740_MOESM1_ESM.pdf]

Supplementary Figures

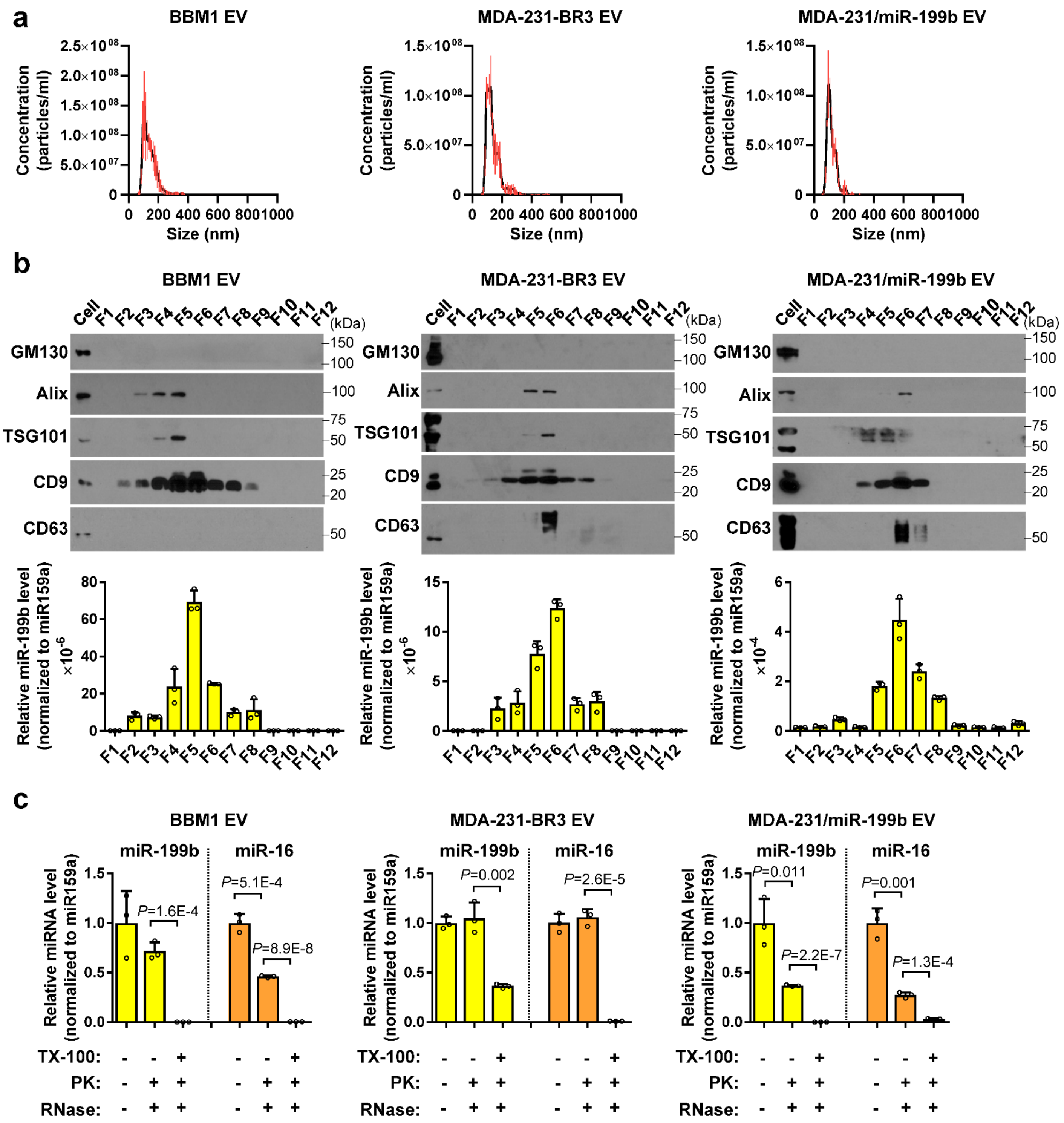

Supplementary Figure 1. miR-199b is encapsulated in EVs secreted by brain-tropic MBC cells. a Nanoparticle tracking analyses of indicated EVs (n=3 biological replicates). **b** Immunoblots of indicated proteins in whole cell lysate and EV fractions from OptiPrep gradient ultracentrifugation showing EV markers and a Golgi marker (GM130, as a negative control for EV-specific proteins). Relative levels of miR-199b in OptiPrep gradient fractions were determined by RT-qPCR and normalized to ath-miR159a spike-in (n=3 biological replicates). **c** Relative levels of miR-199b and miR-16 in EVs treated with Proteinase K (PK, 10 μg/mL) followed by RNase If (RNase, 40 U) or with PBS (as control) in the presence or absence of 1% Triton X-100 (TX-100). RT-qPCR data were normalized to ath-miR159a spike-in added after all treatments (n=3 biological replicates). All values are shown as mean ± SD. Unpaired two-tailed t-test was used in c. P values are indicated. Source data are provided as a Source Data file.

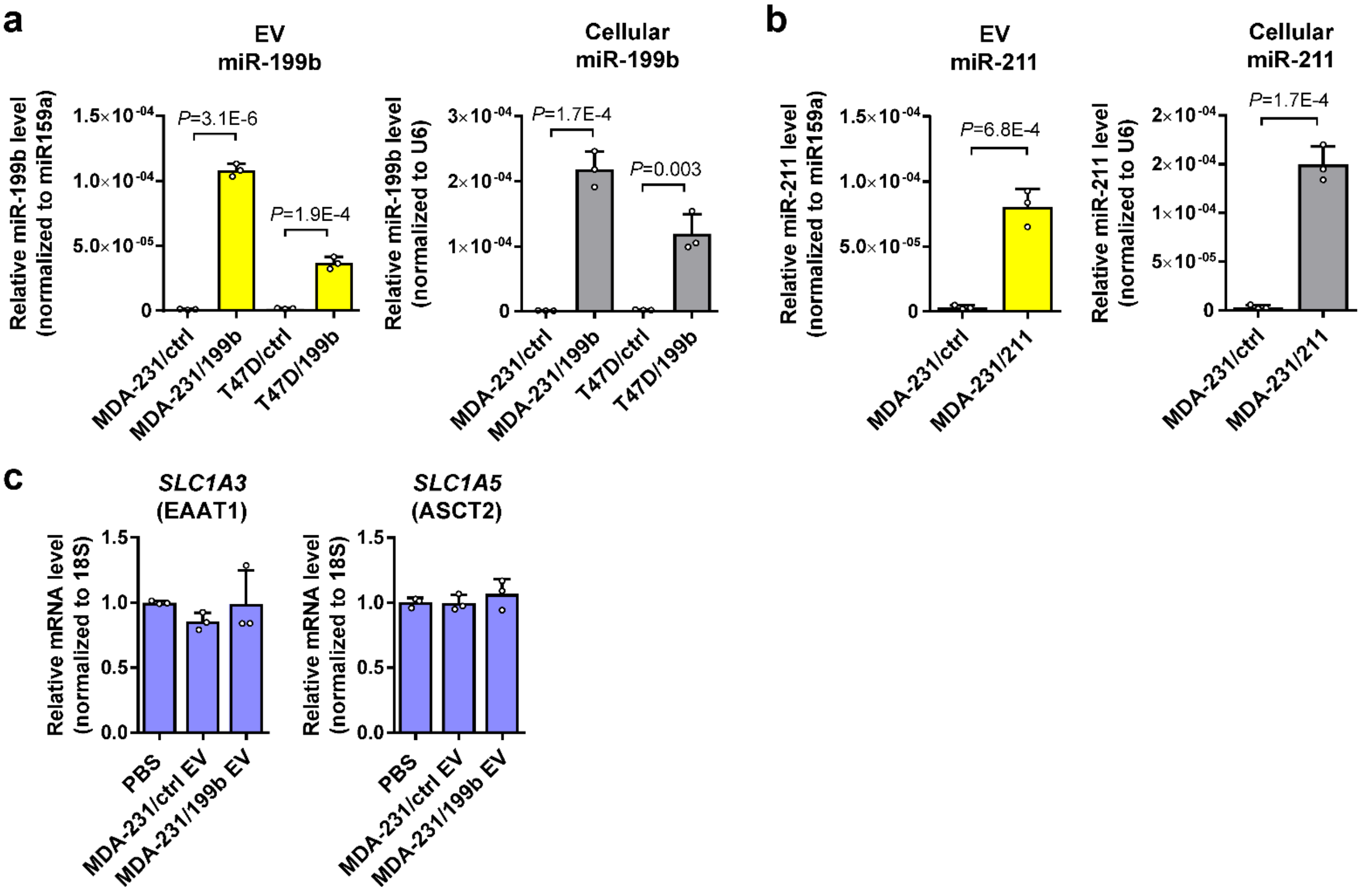

Supplementary Figure 2. Measurements of miRNAs, *SLC1A3* (EAAT1), and *SLC1A5* (ASCT2) expression. **a,b** Relative levels of EV and cellular miRNAs were determined by RT-qPCR, normalized to ath-miR159a spike-in (for EV RNA) or U6 (for cellular RNA), and compared among indicated cell lines (n=3 biological replicates). **c** RT-qPCR-determined RNA levels of *SLC1A3* (EAAT1) and *SLC1A5* (ASCT2) in astrocytes (NHA) upon treatment with indicated EVs. Data were normalized to 18S rRNA (n=3 biological replicates). In bar graphs, values are shown as mean ± SD. Unpaired two-tailed t-test was used in a and b. P values are indicated. One-way ANOVA followed by Tukey's multiple comparison test was used in c, where no statistical significance was found. Source data are provided as a Source Data file.

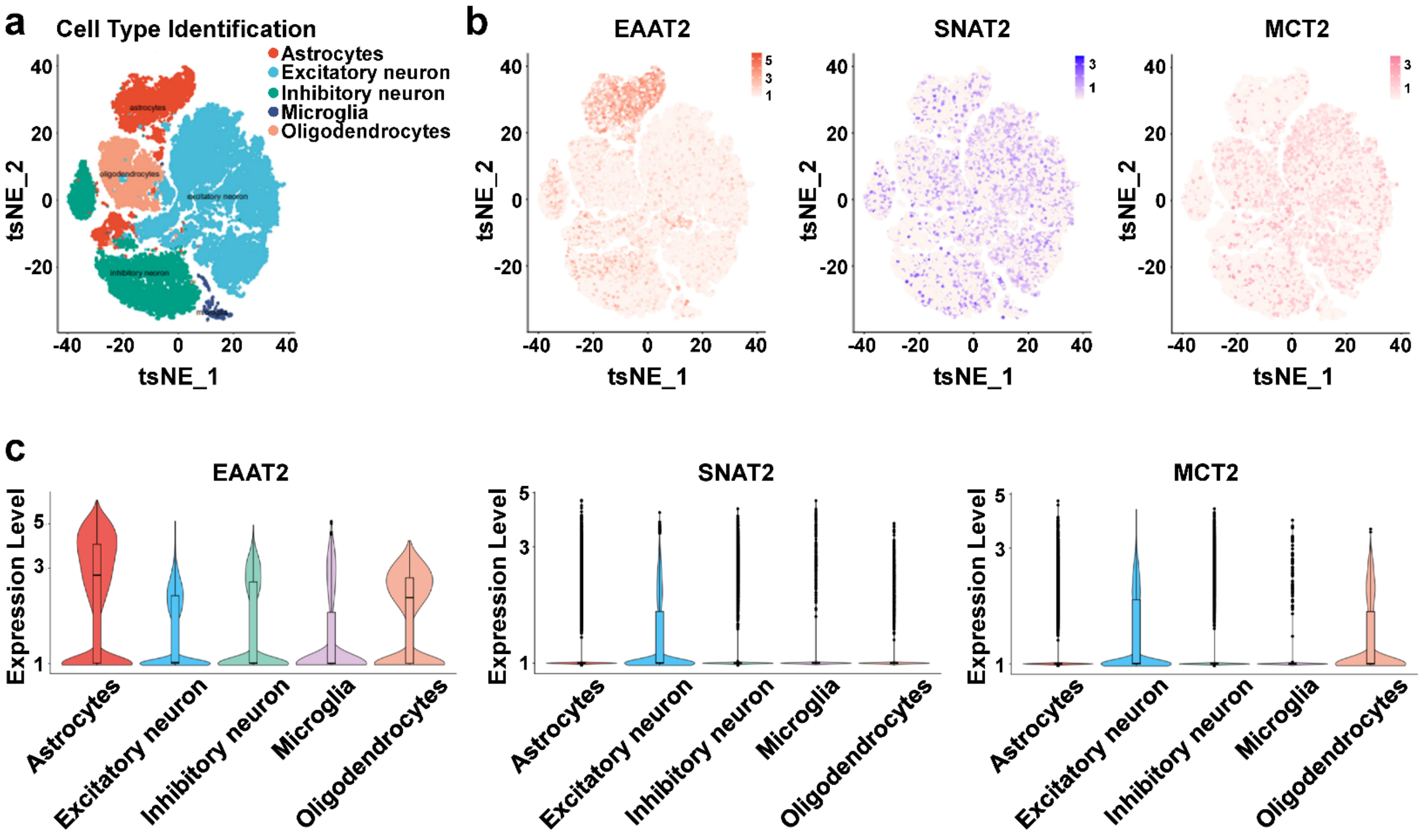

Supplementary Figure 3. The expression patterns of EAAT2, SNAT2 and MCT2 determined by single cell gene expression in the cortex. **a** A t-SNE plot of all high-quality cells colored to indicate the five major cell subsets. **b** Normalized expression levels of EAAT2, SNAT2 and MCT2 in all cell types shown in the t-SNE plot. **c** Vinplots showing gene expression levels of EAAT2, SNAT2 and MCT2 in individual cell clusters.

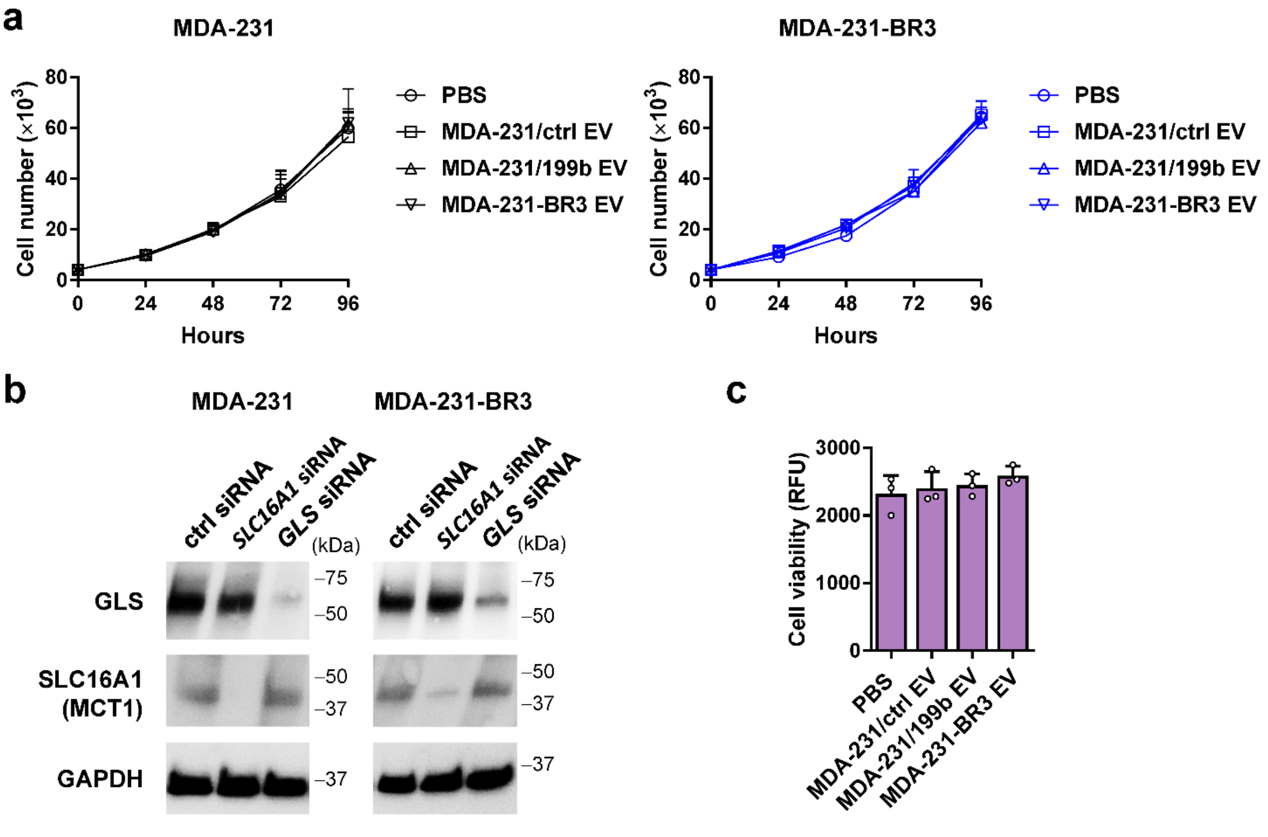

Supplementary Figure 4. *In vitro* cell proliferation, gene knockdown efficiency, and brain slice cell viability. a MDA-231 and MDA-231-BR3 cells pretreated with the indicated EVs for 48 h were seeded onto 12-well plates; EVs were added to the cells every 48 h when the medium was replenished. Cell proliferation was assessed by counting cell numbers every 24 h (n=3 biological replicates). **b** Western blot showing levels of indicated proteins in MDA-231 and MDA-231-BR3 cells transfected with siRNAs for 48 h. GAPDH was used as a sample processing control. The samples derive from the same experiment but different gels for GLS, another for MCT1, and another for GAPDH were processed in parallel. **c** Brain slice cultures from Figure 5c were dissociated and measured for cell viability (n=3 biological replicates). In bar graphs, values are shown as mean ± SD. Two-way ANOVA was used in a (repeated measures), where no statistical significance was found. One-way ANOVA followed by Tukey's multiple comparison test was used in c, where no statistical significance was found. Source data are provided as a Source Data file.

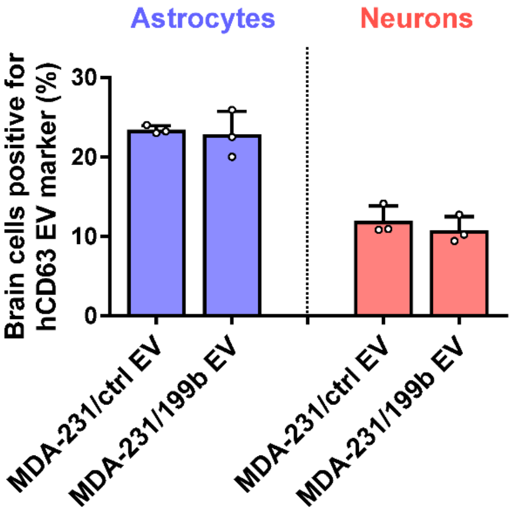

Supplementary Figure 5. Quantification of brain cells positive for the hCD63 EV marker. The fluorescent images of mouse brain sections from Figure 6a were analyzed for the percentage of GFAP$^+$ astrocytes and MAP2$^+$ neurons positive for human-specific CD63, which indicates EV uptake (>100 cells of each type were counted for each brain; n=3 mice per group). In bar graphs, values are shown as mean ± SD. Unpaired two-tailed t-test was used. No statistical significance was found. Source data are provided as a Source Data file.

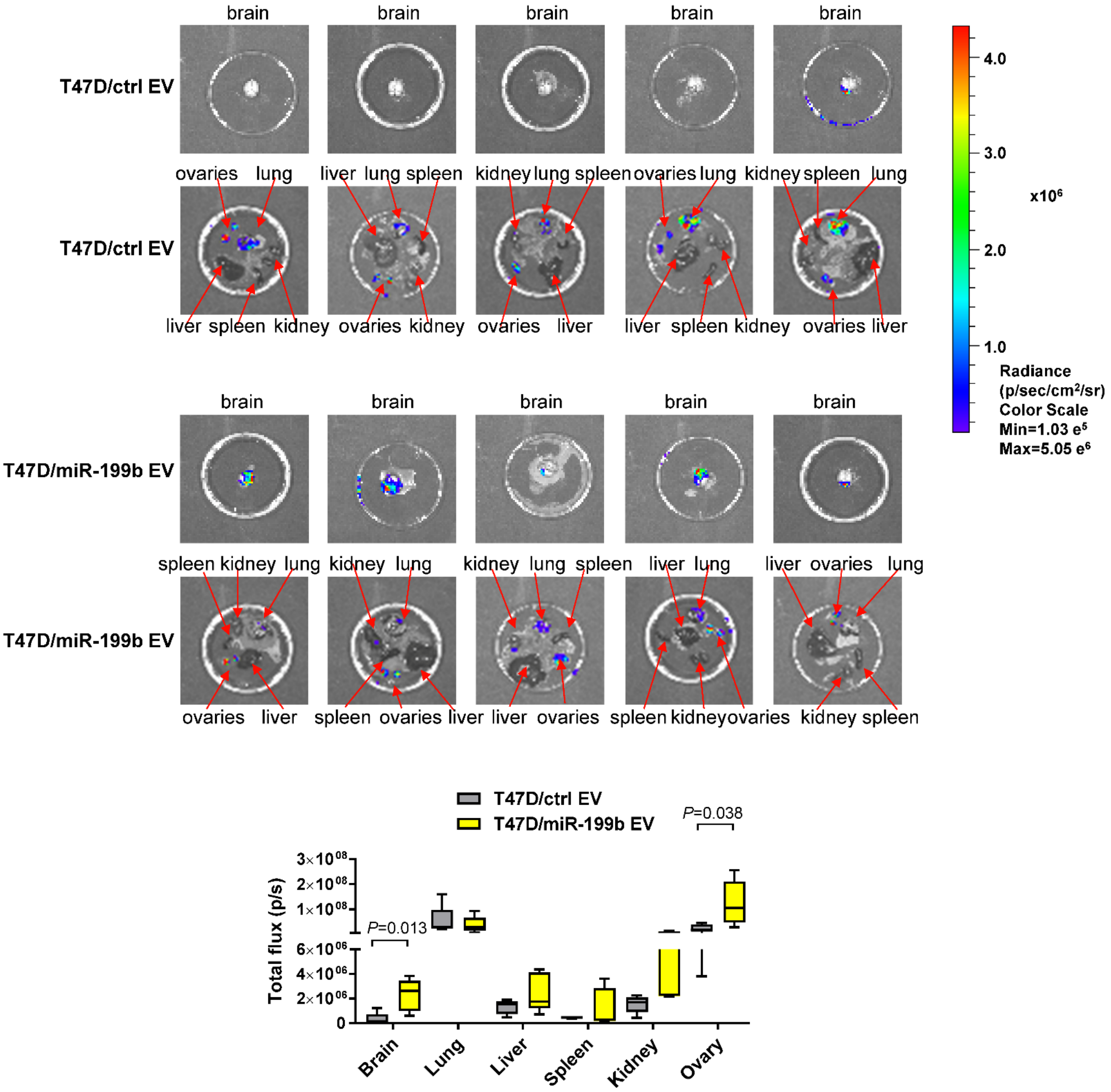

Supplementary Figure 6. Assessments of various organs for T47D-BR2 metastases. Various organs were collected from mice in Figure 7g-i for *ex vivo* bioluminescent imaging and signal quantification (n=5 mice per group). The boxes in the box-and-whiskers plots show the median (centre line) and the quartile range (25–75%), and the whiskers extend from the quartile to the minimum and maximum values. Unpaired two-tailed t-test was used. P values are indicated. Source data are provided as a Source Data file.

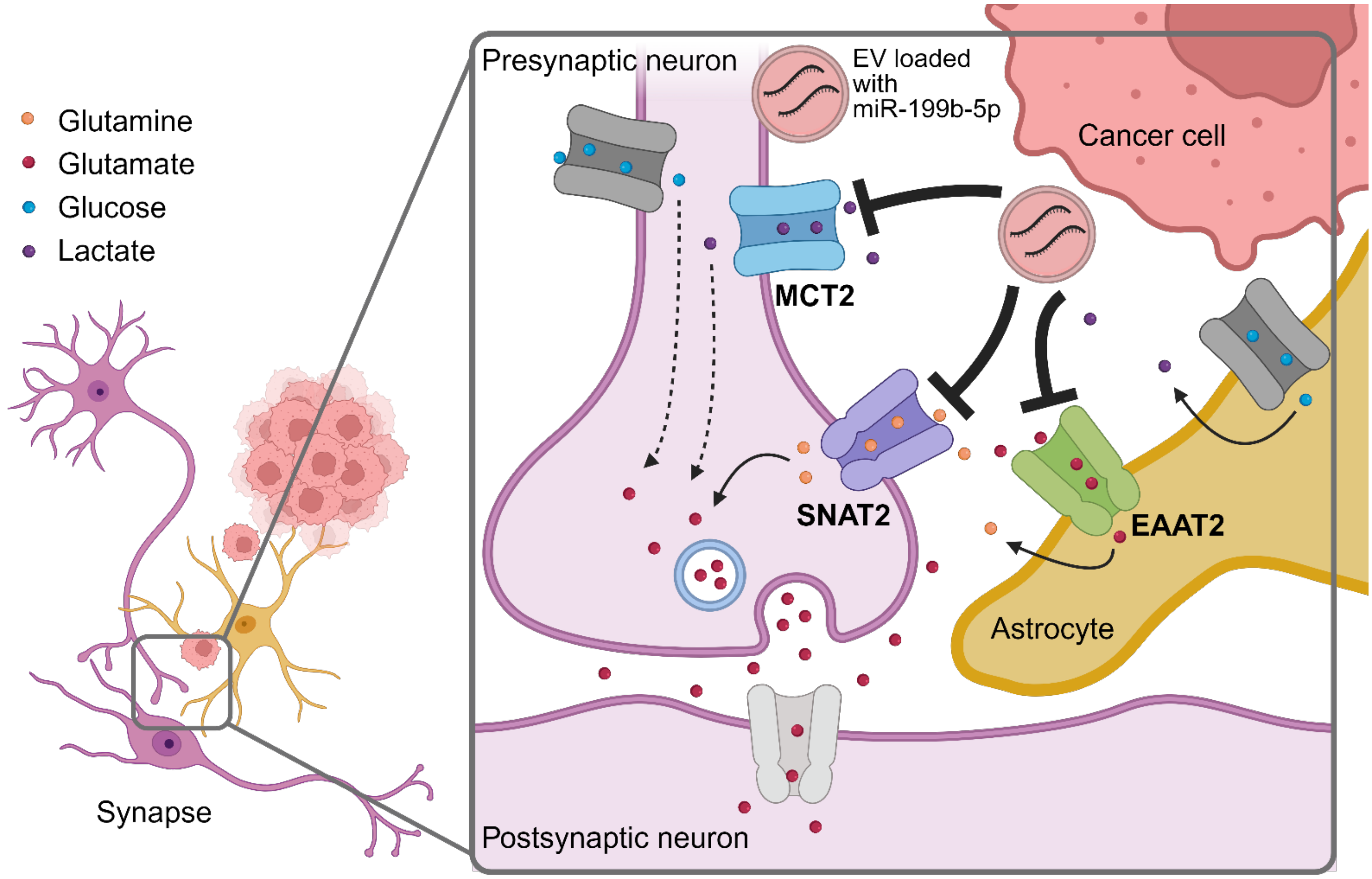

Supplementary Figure 7. A schematic diagram showing the role of BC cell-secreted miR-199b by hijacking neuron–astrocyte metabolic coupling to fuel BC cells metastasized to the brain. Through downregulating membrane transporters controlling influxes of glutamate (EAAT2), glutamine (SNAT2), and lactate (MCT2) into brain cells, BC cells exploit these nutrients in the brain niche to support their expansion. Created with BioRender.com.

Supplementary Table 1: List of primers.

Gene	Forward primer	Reverse primer
**Human *SLC1A2/EAAT2***	CCTGACGGTGTTTGGTGTCAT	CAAGCGGCCACTAGCCTTAG
**Human *SLC38A2/SNAT2***	CCTATGAAATCTGTACAAAAGATTGG	TTGTGTACCCAATCCAAAACAA
**Human *SLC16A7/MCT2***	GGGTTGGATTGTGGTTGGAG	TCCTGCGTACATAACAGCCAG
**Human SLC1A3/EAAT1**	ACATGAAGGAACAGGGGCAG	AGGCACTTGAAGGTGATGGG
**Human SLC1A5/ASCT2**	AAAACCCCTACCGCTTCCTG	GGATGAAACGGCTGATGTGC
**Mouse *Slc1a2/Eaat2***	GCACGAGAGCTATGGTGTATTAC	GTTTGGGATTACCTGGGTGGA
**Mouse *Slc38a2/Snat2***	CAATGAGATCCGTGCAAAAG	TGCTTCCAATCATCACCACT
**Mouse *Slc16a7/Mct2***	AACACAAAGTGGCTAGGCTTAAA	TCACCTGACTGATGTTTCTCTTG
**Human/Mouse *18S rRNA***	CTACCACATCCAAGGAAGGCA	TTTTTCGTCACTACCTCCCCG

Supplementary Table 2: List of antibodies.

Antibody	Source	Identifier	Dilution	Manufacturer's website including validation information
**Anti-EAAT2 antibody**	Abcam	ab41621	1: 1000 (WB)	https://www.abcam.com/eaat2-antibody-ab41621.html
**SLC38A2 Antibody - N-terminal region**	Aviva Systems Biology	ARP33059_P050	1: 1000 (WB)	https://www.avivasysbio.com/slc38a2-antibody-n-terminal-region-arp33059-p050.html
**MCT2 (H-40) for human**	Santa Cruz Biotechnology	sc-50322	1: 500 (WB)	https://datasheets.scbt.com/sc-50322.pdf
**MCT2 (L-11) for mouse**	Santa Cruz Biotechnology	sc-22034-R	1: 500 (WB)	https://datasheets.scbt.com/sc-22034.pdf
**Anti-MAP2 antibody - Neuronal Marker**	Abcam	ab32454	1: 1000 (WB)	https://www.abcam.com/map2-antibody-neuronal-marker-ab32454.html
**GLS1 (E4T9Q) antibody**	Cell Signaling Technology	49363	1: 1000 (WB)	https://www.cellsignal.com/products/primary-antibodies/glutaminase-1-gls1-e4t9q-rabbit-mab/49363
**MCT1/SLC16A1 (E7F6Y) antibody**	Cell Signaling Technology	36768	1: 1000 (WB)	https://www.cellsignal.com/products/primary-antibodies/mct1-slc16a1-e7f6y-rabbit-mab/36768
**GAPDH (D16H11) antibody**	Cell Signaling Technology	5174S	1: 1000 (WB)	https://www.cellsignal.com/products/primary-antibodies/gapdh-d16h11-xp-rabbit-mab/5174
**Alix (E6P9B) Rabbit mAb**	Cell Signaling Technology	92880S	1: 500 (WB)	https://www.cellsignal.com/products/primary-antibodies/alix-e6p9b-rabbit-mab/92880?site-search-type=Products&N=4294956287&Ntt=92880s&fromPage=plp&_requestid=4554038
**TSG101 Polyclonal Antibody**	Invitrogen	PA5-31260	1: 500 (WB)	https://www.thermofisher.com/antibody/product/TSG101-Antibody-Polyclonal/PA5-31260
**Anti-CD63 Antibody (NK1/C3)**	Santa Cruz Biotechnology	sc-59286	1: 500 (WB)	https://www.scbt.com/p/cd63-antibody-nk1-c3
**CD63 Polyclonal antibody**	Proteintech	25682-1-AP	1: 1000 (WB)	https://www.ptglab.com/products/CD63-Antibody-25682-1-AP.htm
**GM130 (D6B1) Rabbit mAb**	Cell Signaling Technology	12480S	1: 1000 (WB)	https://www.cellsignal.com/products/primary-antibodies/gm130-d6b1-xp-rabbit-mab/12480
**CD9 (D3H4P) Rabbit mAb**	Cell Signaling Technology	13403S	1: 1000 (WB)	https://www.cellsignal.com/products/primary-antibodies/cd9-d3h4p-rabbit-mab/13403?site-search-type=Products&N=4294956287&Ntt=13403s&fromPage=plp&_requestid=4555687
**Anti-rabbit IgG, HRP-linked antibody**	Cytiva	NA9340-1ML	1: 8000 (WB)	https://www.fishersci.se/shop/products/anti-rabbit-igg-peroxidase-linked-species-specific-whole-antibody-from-donkey-secondary-antibody-cytiva-formerly-ge-healthcare-life-sciences/10794347
**Anti-mouse IgG, HRP-linked antibody**	Cell Signaling Technology	7076S	1: 5000 (WB)	https://www.cellsignal.com/products/secondary-antibodies/anti-mouse-igg-hrp-linked-antibody/7076

Supplementary Table 3: Clinical characteristics of human specimens.

	Age group at Dx	ER	PR	HER2	Sex
**Case**	50-59	POS	POS	NEG	Female
	40-49	POS	POS	NEG	Female
	40-49	NEG	NEG	NEG	Female
	50-59	NEG	NEG	NEG	Female
	30-39	POS	POS	NEG	Female
	40-49	POS	POS	NEG	Female
	40-49	POS	NEG	POS	Female
	40-49	NEG	NEG	NEG	Female
	30-39	POS	POS	NEG	Female
	60-69	POS	POS	NEG	Female
	50-59	POS	NEG	POS	Female
	40-49	NEG	NEG	POS	Female
	60-69	NEG	NEG	POS	Female
	30-39	POS	POS	POS	Female
	50-59	POS	NEG	NEG	Female
	30-39	POS	POS	POS	Female
	50-59	POS	NEG	POS	Female
	50-59	POS	POS	POS	Female
	50-59	POS	POS	POS	Female
	50-59	NEG	NEG	POS	Female
	60-69	NEG	NEG	NEG	Female
**Control**	30-39	POS	NEG	NEG	Female
	50-59	POS	NEG	NEG	Female
	60-69	POS	POS	POS	Female
	30-39	POS	POS	NEG	Female
	30-39	POS	POS	NEG	Female
	40-49	NEG	NEG	NEG	Female
	50-59	NEG	NEG	POS	Female
	50-59	NEG	NEG	NEG	Female
	40-49	POS	NEG	NEG	Female
	30-39	POS	POS	POS	Female
	60-69	POS	POS	NEG	Female
	50-59	POS	POS	NEG	Female
	30-39	NEG	NEG	POS	Female
	60-69	NEG	NEG	NEG	Female
	60-69	POS	POS	POS	Female
	60-69	NEG	NEG	POS	Female
	40-49	POS	NEG	POS	Female
	40-49	NEG	NEG	POS	Female
	60-69	NEG	NEG	NEG	Female
	50-59	POS	POS	NEG	Female
	40-49	NEG	NEG	POS	Female
