# Supplementary material for: Breast cancer cell-secreted miR-199b-5p hijacks neurometabolic coupling to promote brain metastasis
Source: Nat Commun. 2024 May 29;15:4549. doi: 10.1038/s41467-024-48740-0 (PMC11137082; doi:10.1038/s41467-024-48740-0)
Supplement: Supplementary file 5 — Reporting Summary [file 41467_2024_48740_MOESM5_ESM.pdf]

nature portfolio

Corresponding author(s): Shizhen Emily Wang

Last updated by author(s): Mar 28, 2024

# Reporting Summary

Nature Portfolio wishes to improve the reproducibility of the work that we publish. This form provides structure for consistency and transparency in reporting. For further information on Nature Portfolio policies, see our Editorial Policies and the Editorial Policy Checklist.

## Statistics

For all statistical analyses, confirm that the following items are present in the figure legend, table legend, main text, or Methods section.

| n/a | Confirmed | |
|---|---|---|
| [ ] | [x] | The exact sample size (*n*) for each experimental group/condition, given as a discrete number and unit of measurement |
| [ ] | [x] | A statement on whether measurements were taken from distinct samples or whether the same sample was measured repeatedly |
| [ ] | [x] | The statistical test(s) used AND whether they are one- or two-sided<br>*Only common tests should be described solely by name; describe more complex techniques in the Methods section.* |
| [x] | [ ] | A description of all covariates tested |
| [ ] | [x] | A description of any assumptions or corrections, such as tests of normality and adjustment for multiple comparisons |
| [ ] | [x] | A full description of the statistical parameters including central tendency (e.g. means) or other basic estimates (e.g. regression coefficient) AND variation (e.g. standard deviation) or associated estimates of uncertainty (e.g. confidence intervals) |
| [ ] | [x] | For null hypothesis testing, the test statistic (e.g. *F*, *t*, *r*) with confidence intervals, effect sizes, degrees of freedom and *P* value noted<br>*Give P values as exact values whenever suitable.* |
| [x] | [ ] | For Bayesian analysis, information on the choice of priors and Markov chain Monte Carlo settings |
| [x] | [ ] | For hierarchical and complex designs, identification of the appropriate level for tests and full reporting of outcomes |
| [ ] | [x] | Estimates of effect sizes (e.g. Cohen's *d*, Pearson's *r*), indicating how they were calculated |

*Our web collection on statistics for biologists contains articles on many of the points above.*

## Software and code

Policy information about availability of computer code

| | |
|---|---|
| Data collection | Quantitative PCR (qPCR) data were collected using Bio-Rad CFX Manager (Bio-Rad, version 3.1). IF Images of brain slices captured using a STELLARIS 8 DIVE 2-photon functionality on a DM8 upright microscope (Leica Microsystems, Wetzlar, Germany) were processed using LAS X 4.4 software (Leica Microsystems). Other images captured using a ZEISS LSM 880 confocal microscope system (Carl Zeiss, Oberkochen, Germany) were processed using Image Pro Premier 9.3 software (Media Cybernetics, Rockville, MD). |
| Data analysis | The Ct (threshold cycle) values were obtained by analyzing qPCR data with Bio-Rad CFX Manager (Bio-Rad, version 3.1). Statistical significance was calculated using Prism (GraphPad Software, version 7.01). IF Images of brain slices captured using a STELLARIS 8 DIVE 2-photon functionality on a DM8 upright microscope (Leica Microsystems, Wetzlar, Germany) were processed using LAS X 4.4 software (Leica Microsystems). Other images captured using a ZEISS LSM 880 confocal microscope system (Carl Zeiss, Oberkochen, Germany) were processed using Image Pro Premier 9.3 software (Media Cybernetics, Rockville, MD). 1H-13C correlation spectra were processed using Bruker topspin 3.1, and analyzed with Sparky software (T.D. Goddard and D.G. Kneller, SPARKY 3, University of California, San Francisco). Gene expression data were analyzed using the “SingleR” package (Bioconductor version 3.11). |

For manuscripts utilizing custom algorithms or software that are central to the research but not yet described in published literature, software must be made available to editors and reviewers. We strongly encourage code deposition in a community repository (e.g. GitHub). See the Nature Portfolio guidelines for submitting code & software for further information.

## Data

Policy information about availability of data

All manuscripts must include a data availability statement. This statement should provide the following information, where applicable:

- Accession codes, unique identifiers, or web links for publicly available datasets
- A description of any restrictions on data availability
- For clinical datasets or third party data, please ensure that the statement adheres to our policy

Source data are provided with this paper. Small RNA-seq data that support the findings of this study are deposited in the Gene Expression Omnibus (GEO) under accession code GSE216934 (https://www.ncbi.nlm.nih.gov/geo/query/acc.cgi?acc=GSE216934). Dataset GSE168408 (https://www.ncbi.nlm.nih.gov/geo/query/acc.cgi?acc=GSE168408) is re-analyzed in this study. The NMR data generated in this study have been deposited in the Zenodo database under accession code DOI: 10.5281/zenodo.10927179 (https://doi.org/10.5281/zenodo.10927179). All other data supporting the findings of this study are available from the corresponding author on reasonable request.

## Research involving human participants, their data, or biological material

Policy information about studies with human participants or human data. See also policy information about sex, gender (identity/presentation), and sexual orientation and race, ethnicity and racism.

| | |
|---|---|
| Reporting on sex and gender | All 42 patients involved in this study were females with stage IV breast cancer disease. All participants provided written informed consent. |
| Reporting on race, ethnicity, or other socially relevant groupings | The study did not involve classifications based on race, ethnicity, or other socially relevant groupings. The genetic ancestry data were not available to researchers. |
| Population characteristics | All 42 patients involved in this study were females with stage IV disease either with or without brain metastases at the time metastatic disease was diagnosed. Among them, 21 patients had brain metastases (case), in some cases with concurrent metastases to other organs, whereas the other 21 patients (control) had distant metastases to other organs without the involvement of central nervous system. The two groups exhibited balanced age, tumor subtype, and sample collection time. Serum specimens examined in this study were collected at the time metastasis was initially diagnosed or the earliest draw available. The case group has an average age of 49.7+/-10.3 years with positive ER, PR, and HER2 respectively detected in 14, 10, and 10 tumors. The control group has an average age of 50.0+/-11.2 years, with positive ER, PR, and HER2 respectively detected in 12, 8, and 9 tumors. |
| Recruitment | Archived samples from cancer patients used in this study were collected in accordance with the Declaration of Helsinki and the principles of Good Clinical Practice. All participants provided written informed consent. Human serum specimens were obtained from voluntarily consenting breast cancer patients between February 2006 and December 2011 at the City of Hope National Medical Center (Duarte, CA, USA) under institutional review board-approved protocols. |
| Ethics oversight | Human serum specimens were obtained from voluntarily consenting breast cancer patients between February 2006 and December 2011 at the City of Hope National Medical Center (Duarte, CA, USA) under institutional review board-approved protocols. |

Note that full information on the approval of the study protocol must also be provided in the manuscript.

# Field-specific reporting

Please select the one below that is the best fit for your research. If you are not sure, read the appropriate sections before making your selection.

☒ Life sciences ☐ Behavioural & social sciences ☐ Ecological, evolutionary & environmental sciences

For a reference copy of the document with all sections, see nature.com/documents/nr-reporting-summary-flat.pdf

# Life sciences study design

All studies must disclose on these points even when the disclosure is negative.

| | |
|---|---|
| Sample size | Sample size was generally chosen based on preliminary data indicating the variance within each group and the differences between groups. |
| Data exclusions | No samples or animals were excluded from the analysis. |
| Replication | Except for small RNA-seq, all other experiments were performed at least twice independently with similar results. Relative levels of the miRNAs selected from small RNA-seq results were verified by RT-PCR, and consistent results were obtained. |
| Randomization | All mice/samples were randomized before experiments. |
| Blinding | Data collection and analysis were performed blinded to group allocation. |

# Reporting for specific materials, systems and methods

We require information from authors about some types of materials, experimental systems and methods used in many studies. Here, indicate whether each material, system or method listed is relevant to your study. If you are not sure if a list item applies to your research, read the appropriate section before selecting a response.

## Materials & experimental systems

| n/a | Involved in the study |
|---|---|
| | ☒ Antibodies |
| | ☒ Eukaryotic cell lines |
| ☒ | ☐ Palaeontology and archaeology |
| | ☒ Animals and other organisms |
| ☒ | ☐ Clinical data |
| ☒ | ☐ Dual use research of concern |
| ☒ | ☐ Plants |

## Methods

| n/a | Involved in the study |
|---|---|
| ☒ | ☐ ChIP-seq |
| ☒ | ☐ Flow cytometry |
| ☒ | ☐ MRI-based neuroimaging |

## Antibodies

Antibodies used

Anti-EAAT2 antibody (Abcam ab41621); SLC38A2 Antibody - N-terminal region (Aviva Systems Biology ARP33059_P050); MCT2 (H-40) to detect human MCT2 (Santa Cruz Biotechnology sc-50322); MCT2 (L-11) to detect mouse MCT2 (Santa Cruz Biotechnology sc-22034-R); Anti-MAP2 antibody - Neuronal Marker (Abcam ab32454); GLS1 (E4T9Q) antibody (Cell Signaling Technology 49363); MCT1/SLC16A1 (E7F6Y) antibody (Cell Signaling Technology 36768); GAPDH (D16H11) Rabbit mAb (Cell Signaling Technology 5174S); Alix (E6P9B) Rabbit mAb (Cell Signaling Technology 92880S); TSG101 Polyclonal Antibody (Invitrogen PA5-31260); Anti-CD63 Antibody (NK1/C3) (Santa Cruz Biotechnology sc-59286); CD63 Polyclonal antibody (Proteintech 25682-1-AP); GM130 (D6B1) Rabbit mAb (Cell Signaling Technology 12480S); CD9 (D3H4P) Rabbit mAb (Cell Signaling Technology 13403S); Anti-rabbit IgG, HRP-linked antibody (Cytiva NA9340-1ML); Anti-mouse IgG, HRP-linked antibody (Cell Signaling Technology 7076S)

Validation

Antibodies are validated according to the manufacturer's instructions.
Anti-EAAT2 antibody 1: 1000 (WB) https://www.abcam.com/eaat2-antibody-ab41621.html
SLC38A2 Antibody - N-terminal region 1: 1000 (WB) https://www.avivasysbio.com/slc38a2-antibody-n-terminal-region-arp33059-p050.html
MCT2 (H-40) to detect human MCT2 1: 500 (WB) https://datasheets.scbt.com/sc-50322.pdf
MCT2 (L-11) to detect mouse MCT2 1: 500 (WB) https://datasheets.scbt.com/sc-22034.pdf
Anti-MAP2 antibody - Neuronal Marker 1: 1000 (WB) https://www.abcam.com/map2-antibody-neuronal-marker-ab32454.html
GLS1 (E4T9Q) antibody 1: 1000 (WB) https://www.cellsignal.com/products/primary-antibodies/glutaminase-1-gls1-e4t9q-rabbit-mab/49363
MCT1/SLC16A1 (E7F6Y) antibody 1: 1000 (WB) https://www.cellsignal.com/products/primary-antibodies/mct1-slc16a1-e7f6y-rabbit-mab/36768
GAPDH (D16H11) antibody 1: 1000 (WB) https://www.cellsignal.com/products/primary-antibodies/gapdh-d16h11-xp-rabbit-mab/5174
Alix (E6P9B) Rabbit mAb 1: 500 (WB) https://www.cellsignal.com/products/primary-antibodies/alix-e6p9b-rabbit-mab/92880?site-search-type=Products&N=4294956287&Ntt=92880s&fromPage=plp&_requestid=4554038
TSG101 Polyclonal Antibody 1: 500 (WB) https://www.thermofisher.com/antibody/product/TSG101-Antibody-Polyclonal/PA5-31260
Anti-CD63 Antibody (NK1/C3) 1: 500 (WB) https://www.scbt.com/p/cd63-antibody-nk1-c3
CD63 Polyclonal antibody 1: 1000 (WB) https://www.ptglab.com/products/CD63-Antibody-25682-1-AP.htm
GM130 (D6B1) Rabbit mAb 1: 1000 (WB) https://www.cellsignal.com/products/primary-antibodies/gm130-d6b1-xp-rabbit-mab/12480
CD9 (D3H4P) Rabbit mAb 1: 1000 (WB) https://www.cellsignal.com/products/primary-antibodies/cd9-d3h4p-rabbit-mab/13403?site-search-type=Products&N=4294956287&Ntt=13403s&fromPage=plp&_requestid=4555687
Anti-rabbit IgG, HRP-linked antibody 1: 8000 (WB) https://www.fishersci.se/shop/products/anti-rabbit-igg-peroxidase-linked-species-specific-whole-antibody-from-donkey-secondary-antibody-cytiva-formerly-ge-healthcare-life-sciences/10794347
Anti-mouse IgG, HRP-linked antibody 1: 5000 (WB) https://www.cellsignal.com/products/secondary-antibodies/anti-mouse-igg-hrp-linked-antibody/7076

## Eukaryotic cell lines

Policy information about cell lines and Sex and Gender in Research

Cell line source(s)

MCF-10A (CRL-10317; female), MDA-MB-231 (HTB-26; female), T47D (HTB-133; female), and SH-SY5Y (CRL-2266; female) cells were obtained from American Type Culture Collection (ATCC; Manassas, VA). MCF-10A cells were cultured as described91. MDA-MB-231 cells were cultured in Dulbecco's Modified Eagle's medium (DMEM; Gibco, Waltham, MA) supplemented with 10% fetal bovine serum (FBS; Sigma-Aldrich, St. Louis, MO). T47D cells were cultured in RPMI-1640 medium (Gibco) supplemented with 10% FBS. The brain-tropic subline MDA-MB-231-BR3 was developed from the MDA-231BR brain-seeking clone previously generated by Dr. Yoneda et al. To further enhance brain tropism, MDA-231BR cells were inoculated into the left ventricle of the heart in female NSG mice and brain metastases were isolated and cultured in vitro to generate MDA-MB-231-BR2, which subsequently underwent another round of in vivo selection and explant culture

| | |
|---|---|
| | to generated MDA-MB-231-BR3 from the brain metastases. The bone-tropic subline of MDA-MB-231 (MDA-231-Bone) was previously generated by Dr. Yoneda et al. and used in our previous study. The lung-metastatic MDA-MB-231 (MDA-231-LM2) was a gift from Drs. Yibin Kang and Hanqiu Zheng. A brain-tropic subline of T47D, namely T47D-BR2, was generated from a spontaneous brain metastasis of T47D using the same in vivo selection strategy and was a gift from Dr. Yumei Feng. SH-SY5Y cells were maintained in DMEM/F-12 supplemented with 10% FBS and antibiotic-antimycotic (Gibco). Differentiation was induced by culturing SH-SY5Y cells in DMEM/F-12 supplemented with 2% FBS, antibiotic-antimycotic, and 10 µM all trans-retinoic acid (Sigma-Aldrich) for 7 days. A neuron-like phenotype was confirmed by expression of MAP2, a neuronal marker, by Western blotting. Normal human astrocytes (NHA; female; lot # 0000565612) were obtained from Lonza (Basel, Switzerland) and cultured in astrocyte basal medium with SingleQuots supplements (Lonza) following manufacturer's instructions. Patient-derived breast-to-brain metastatic (BBM) cells (female) were developed by Dr. Jandial's group from resected specimens as described previously. |
| Authentication | short tandem repeat profiling |
| Mycoplasma contamination | All cells used herein were tested to be free of mycoplasma contamination. |
| Commonly misidentified lines (See ICLAC register) | No cell lines used in this study were found in the database of commonly misidentified cell lines that is maintained by ICLAC and NCBI Biosample. |

## Animals and other research organisms

Policy information about studies involving animals; ARRIVE guidelines recommended for reporting animal research, and Sex and Gender in Research

| | |
|---|---|
| Laboratory animals | Female NOD/SCID/IL2Rγ-null (NSG) mice of ~8-week-old were used in all experiments except for preparation of brain slices, in which female C57BL/6 mice of ~8-week-old were used. |
| Wild animals | This study did not involve wild animals. |
| Reporting on sex | All cells and clinical specimens used in this study were female, as ~99% of breast cancer occurs in women. |
| Field-collected samples | This study did not involve field-caught samples. |
| Ethics oversight | All animal experiments were approved by the institutional animal care and use committee (IACUC) at the University of California San Diego and City of Hope Beckman Research Institute. |

Note that full information on the approval of the study protocol must also be provided in the manuscript.

## Plants

| | |
|---|---|
| Seed stocks | n/a |
| Novel plant genotypes | n/a |
| Authentication | n/a |
